# Nanoparticles as Drug Delivery Systems of RNAi in Cancer Therapy

**DOI:** 10.3390/molecules26082380

**Published:** 2021-04-19

**Authors:** Diedie Li, Chengzhi Gao, Meiyan Kuang, Minhao Xu, Ben Wang, Yi Luo, Lesheng Teng, Jing Xie

**Affiliations:** 1 School of Pharmacy and Bioengineering, Chongqing University of Technology, Chongqing 400054, China; ldd@2018.cqut.edu.cn (D.L.); ChengzhiGao@2019.cqut.edu.cn (C.G.); kmy72@2020.cqut.edu.cn (M.K.); xuminhao@2020.cqut.edu.cn (M.X.); WB469600102@2020.cqut.edu.cn (B.W.); Luoyi123@2020.cqut.edu.cn (Y.L.); 2 School of Life Sciences, Jilin University, Changchun 130012, China; tenglesheng@jlu.edu.cn

**Keywords:** nanoparticles, RNA interference, cancer

## Abstract

RNA interference (RNAi) can mediate gene-silencing by knocking down the expression of a target gene via cellular machinery with much higher efficiency in contrast to other antisense-based approaches which represents an emerging therapeutic strategy for combating cancer. Distinct characters of nanoparticles, such as distinctive size, are fundamental for the efficient delivery of RNAi therapeutics, allowing for higher targeting and safety. In this review, we present the mechanism of RNAi and briefly describe the hurdles and concerns of RNAi as a cancer treatment approach in systemic delivery. Furthermore, the current nanovectors for effective tumor delivery of RNAi therapeutics are classified, and the characteristics of different nanocarriers are summarized.

## 1. Introduction

Cancer is still defined as a major public health problem in the world [1]. Traditional cancer treatments, such as chemotherapy and radiation therapy, may result in toxicity to normal organs and tissues due to the fact of their non-targeting properties. Since the conversion of normal cells to malignant cells is associated with the deregulation of gene expression, emerging cancer treatment strategies, such as gene therapy, are gaining a lot of attention.

A report by Andrew Fire et al. [2], published in 1998, showed that double-stranded RNA led to more effective interference effects compared with single-stranded RNA; this is the oldest finding demonstrating the significance of double-stranded RNA for RNA interference. Then, Sayda M. Elbashir et al. [3] found that the expression of endogenous and heterologous genes could be efficiently inhibited in cultured mammalian cells by using 21-nucleotide siRNA duplexes in 2001. It is widely accepted that every gene related to disease has the potential to become the target of siRNA, which can easily inhibit the expression of any gene via a base sequence alone [4]. Over the past 20 years, significant progress has been made in the clinical application of RNAi therapy due to the efforts, including financial resources and manpower, made by researchers. It is particularly worth mentioning that in 2018, the first RNAi-based therapeutic drug patisiran (Onpattro^®^), a lipid-based system aimed at triggering TTR gene silencing in patients with hereditary transthyretin-mediated amyloidosis (hATTR amyloidosis), was approved by the FDA, which is promising news [5,6].

Remarkable advances in molecular and cell biology pave the way for the application of RNAi-mediated gene silencing in cancer treatment [7]. RNA interference regulates the cancer-relevant target gene including those that are difficult to address with conventional therapeutics and holds the promise for developing new cancer treatment drugs with lower toxicity and higher selectivity. An RNAi-based therapeutic can offer a new paradigm for anti-cancer intervention in contrast to other inhibitors. However, naked siRNA can easily trigger an innate immune response and be degraded by ribonucleases. Moreover, siRNA is negatively charged and the molecular weight of it is too large (~13 kDa) so that it is difficult for siRNA to cross cellular membranes. As a consequence of its inherent properties, the issues of non-toxicity and effective delivery are thought to be the most significant barriers between RNAi technology and its clinical application. In comparison to other carriers, nanoparticles provide unique benefits and have great potential to serve as the shield for the effective delivery of siRNA in the circulatory system. In 2014, Atu027, a lipid-based RNAi therapeutic aimed against the protein kinase N3 (PKN3) mRNA in the vascular endothelium to treat pancreatic ductal adenocarcinoma (PDAC), was well tolerated in a dose–escalation phase I clinical trial. More than 90% of Atu027 adverse events were limited to Grade 1 or 2, showing great safety [8]. In addition, another lipid-based siRNA nanoparticle, termed DCR-MYC, was developed to downregulate the MYC, which is an oncoprotein that is deregulated in most malignancies. In a dose–escalation phase I clinical trial, patients with different tumor types, including neuroendocrine tumor (NET), metastatic breast cancer (MBC), colorectal cancer (CRC) or others, were treated with DCR-MYC across five dose levels. Tumor shrinkage was observed in multiple patients after treatment [9]. All those results suggest that nanoparticle-based RNAi approaches are a promising avenue for cancer treatment. Table 1 details the RNAi-based cancer therapies in clinical trials.

## 2. The Mechanism of RNA Interference

**Figure 1:**
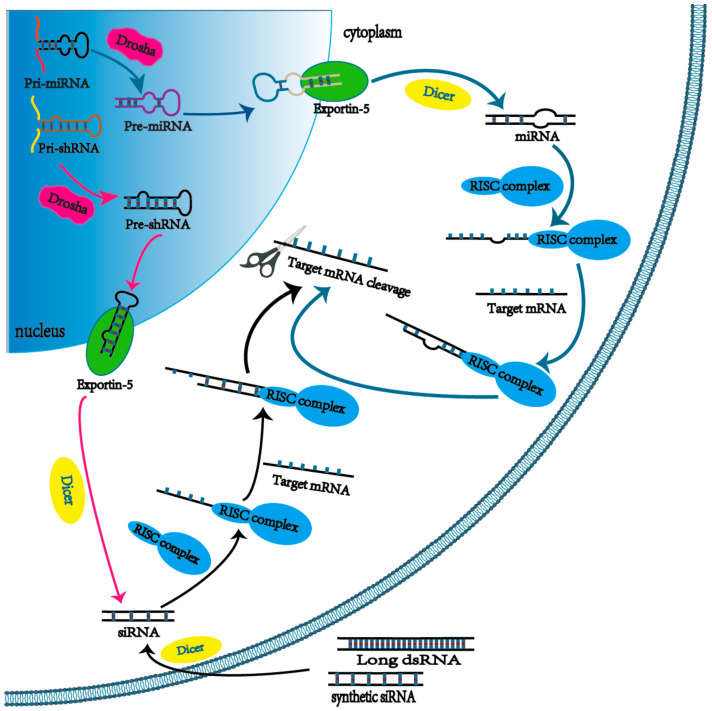
The mechanism behind RNAi.

One pathway of RNAi begins with mature siRNA in the perinuclear region of the cytoplasm [22]. In detail, foreign double-stranded RNAs (dsRNA) are typically excised by Dicer, an endoribonuclease or helicase with RNase motif, into 21–24 nucleotides, termed mature siRNA. Then, the guide strand binds to the RISC to mediate the gene silencing activity, while the non-guiding strand of the mature siRNA is degraded by Argonaute 2 (Ago2). Subsequently, the guide strand in complex with RISC searches the target mRNA according to the Watson–Crick base pairing principle. Once located, the Ago2 protein in RISC cleaves the target mRNA for degradation, at which point the ability of target genes to express proteins is inhibited. Unlike to antisense oligonucleotides, RISC can facilitate multiple rounds of target mRNA cleavage so that siRNA is more effective in silencing effect. Another pathway commences with the transcription of microRNAs (miRNA), which have been studied as a significant regulator of gene expression in recent years. The miRNAs are transcribed from the host genome by RNA polymerase II as a primary miRNA (pri-miRNA) and then processed by a protein complex containing the Drosha into 65–70 nucleotide hairpin-like pre-miRNA (precursor miRNA). It is then transported from the nucleus through exportin-5 (Exp5), a specialized nuclear membrane protein, to the cytoplasm. Here, pre-miRNAs also can be processed by Dicer to form 18–25 nucleotide mature miRNAs that can bind to RISC, leading the mRNA cleavage and translation repression (Figure 1). Unlike the siRNA pathway, not all nucleotide sequences of the mature miRNA can bind to the target mRNA due to the fact of its specific non-linear structure. Thus, individual miRNAs usually have several different target mRNAs in the regulation of gene expression. On the other hand, short hairpin RNA (shRNA), a nucleotide sequence in the nucleus from the transcription of a DNA vector, can facilitate long-term gene silencing via RNAi, since the shRNA can be synthesized by the host cell continuously [23]. In detail, the primary transcript, pri-shRNA, is processed by Drosha to form a structure with a 50–70 nucleotide long loop-stem called pre-shRNA and then transported to the cytoplasm by Exp-5 to mediate gene silencing via a pathway similar to the synthetic siRNA. The general properties between siRNA, miRNA, and shRNA are illustrated in Table 2.

## 3. Limitations of RNAi as a Therapeutic Approach in Systemic Delivery

### 3.1. Modes of Administration

Due to the inherent properties of siRNA, such as being negatively charged and easily degraded by nucleases, efficient delivery of siRNA to the target sites is a serious challenge. Local administration, such as nasal sprays and eye drops, have been used to deliver siRNA into the tissues that are external or readily accessible. Intravitreal injection of naked siRNA with VEGF mRNA as the target is one of the earliest clinical trials of siRNA local administration [4]. However, Bevasiranib, a 21 mer siRNA developed by Opko Health Inc. ended with disappointing results due to the poor efficacy in reducing vision loss in Phase III clinical trials [24]. In the vast majority of human diseases, many important target sites are not directly accessible so that they can only be reached via systemic administration of RNAi therapeutics instead of topical administration [25].

### 3.2. Renal Clearance and Size Dependency

Elimination by the kidneys leading to a short half-life and poor efficacy is a problem in systemic administration of siRNA. A study related to biodistribution of siRNA in rats conducted by Femke M. van de Water et al. [26], in 2006, showed that after intravenous administration, siRNA mainly accumulated in the kidney and was excreted in the urine. Thus, the kidney, an organ for blood filtration and excretion of waste, is of vital importance in the transport and clearance of siRNA drugs in vivo. However, the kidney filtration effect can be effectively avoided while the hydrodynamic diameter of an siRNA drug administered intravenously into the circulatory system is greater than 6 nm [27]. Thus, a strategy to enhance the residence time of siRNA in the circulatory system is to appropriately increase the particle size of siRNA delivery nanoparticles via a proper modification or formulation.

### 3.3. Nuclease Degradation and Immune System Recognition

Degradation by endogenous nucleases has always been a concern when an intravenous siRNA therapeutic is navigating in the bloodstream. As a consequence of its instability, the half-life of intravascular naked siRNA is very short [28]. In detail, nucleases bind to RNAs and attack their unstable ends to generate fragments for degradation [22]. Given the high activity of ribonucleases and inherent characteristics of siRNAs, a shield to prevent siRNA from degradation is necessary. The siRNA therapy is further limited because certain motifs in siRNA oligo can mediate innate immune response by Toll-like receptor 3, 7, and 8 [29,30]. To reduce the potential toxicity of RNAi therapy and limit the uptake by immune cells, a modification for siRNA delivery nanoparticles such as pegylation has been used to help siRNA nanoparticles escape the uptake of phagocytes [31]. It is well established that the design of siRNA delivery strategies to evade the recognition of the immune system is important.

### 3.4. Heterogeneity of Tumor Vasculature

In order to accumulate in the tumor microenvironment (TME), the nanoparticles-based siRNA complex must move through the extracellular matrix (ECM), a dense network of fibrous proteins and polysaccharides, after leaving the bloodstream [32]. Given the compactness of the ECM network, it can obstruct the transportation of nanoparticles. The highly developed ECM hinders the diffusion of nanoparticles through the interstitium; thus, part of the nanoparticles cannot have its intended treatment effect. Abnormal vascular structure, aberrant blood flow, and lack of lymphatic drainage are the unique characteristics of tumor tissue [33]. Thus, nanoparticles will passively accumulate in tumors to a greater extent compared to the normal tissue, when ranging in size from 30 to 200 nm, thereby leading to enhance of the therapeutic index, termed the EPR effect [33,34]. Due to the inherent characteristics of tumor vasculature, not every region of the tumor shares the same concentration of nanoparticles, so that despite the concentration of nanoparticles in tumors being able to be increased through EPR’s effects, complete eradication of tumors is still a difficult problem. Moreover, for solid tumors, the permeability of vessels may be different and the EPR effect may not be exhibited, so the passive targeting strategy may be subject to many limitations [35]. However, these limitations can be overcome by binding the targeting ligands, such as antibodies and their fragments and peptides, to the surface of siRNA-delivering nanoparticles [36].

### 3.5. Endosomal Escape

The siRNA-delivery vectors that have successfully arrived at the periphery of the target cell must be internalized into the cytoplasm in which even further barriers await. Endocytosis that occurs at the cell surface is the major cellular uptake mechanism for any biological agents including siRNA [37,38]. Once endocytosed, the endocytic vesicles containing siRNA transported in early endosomal vesicles and eventually fuse with the lysosomes containing a variety of nucleases and a more acidic environment (pH 4.5–5). After a long journey to reach the target cancer cells, a large portion of the siRNA will be degraded instead of being released to mediate the RNAi pathway [39]. Thus, one of the major obstacles to achieving an effective siRNA therapy is the lack of an endosomal escape strategy. In order to avoid degradation by lysosomes as much as possible, many nanocarriers that can facilitate the endosomal escape have been developed. Several chemical agents have buffering capacities under certain conditions, such as polyethylenimine (PEI) in the range of pH 5–8, which can help to improve endosomal escape via the proton sponge effect [40]. Moreover, chloroquine can contribute to endosomal escape by increasing the pH of the endosome environment to disrupt the endosomal membrane. However, due to the fact of its toxicity, it is impractical to apply chloroquine to in vivo siRNA delivery [41]. Additionally, other agents that may be useful to enhance endosomal escape, such as proteins and peptides, can be included in nanoparticle formulations [38].

## 4. Protective Carriers for siRNA Delivery

Given the intracellular and extracellular barriers of siRNA therapeutics, it is necessary to develop a multi-functional vector to facilitate the safe and effective delivery of siRNA to tumor cells. In recent years, nanoparticles, as the emerging protective delivery platforms for siRNA, have attracted a lot of attention. In this part, RNAi delivery systems based on nanoparticles are categorized and their properties are summarized.

### 4.1. Lipid-Based Nanoparticles

In the research of nanotechnology, many efforts are trying to create artificial membranes, which are similar to cytomembranes in structures and functions. Naturally, lipid-based nanoparticles emerged in various studies [42]. Lipid nanoparticles are composed of one or more lipid bilayers, discovered first by British scientist Bangham in the 1960s [43]. The hydrophilic head of the phospholipid molecule is extended to the aqueous solution, while lipophilic drugs can be loaded into the hydrophobic core of the lipid nanoparticles [44,45]. The structure of lipid nanoparticles resembles the vesicles of cytomembrane [46,47]. Due to the special structure and properties, people quickly realized the potential value of lipid materials in drug delivery. With the development, nanoparticles made of lipids have become the most commonly used material for siRNA delivery to tumor sites with the proportion up to 25% [48]. Up to now, commonly used lipid nanoparticles include liposomes and solid lipid nanoparticles (SLNs).

#### 4.1.1. Liposomes

**Figure 2:**
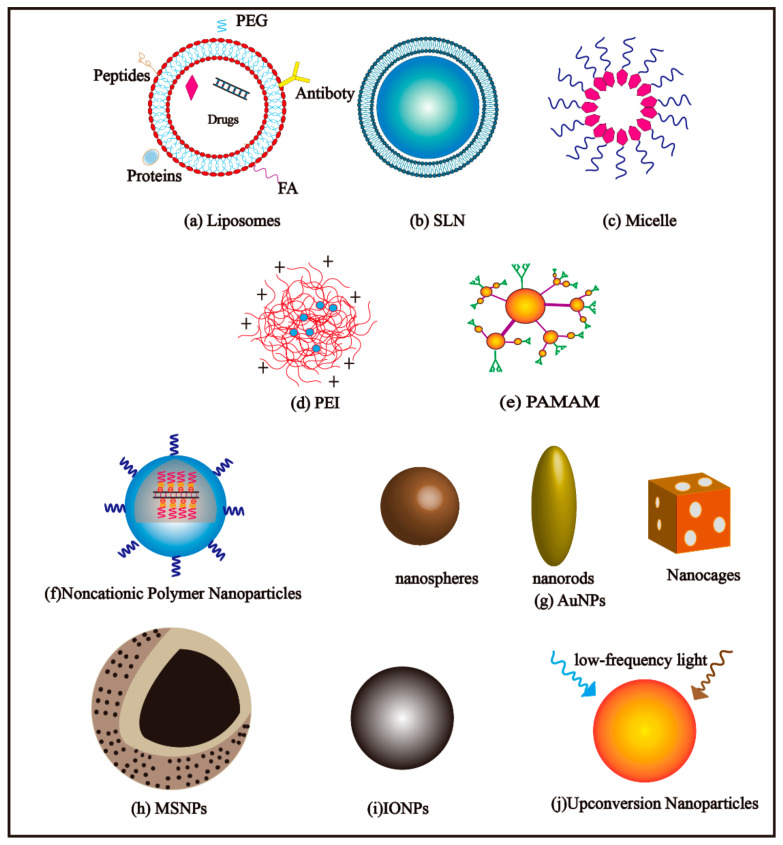
Schematic images of each representative nanoparticle delivery system. (**a**) Liposomes; (**b**) SLN; (**c**) Micelle; (**d**) PEI; (**e**) PAMAM; (**f**) Noncationic Polymer Nanoparticles; (**g**) AuNPs; (**h**) MSNPs; (**i**) IONPs; (**j**) Upconversion Nanoparticles.

For liposomes (Figure 2a), while they are widely used to deliver chemical drugs, genes, and siRNAs (small interfering RNAs) [49], it has low mechanical stability due to the fact of their small membrane thickness, and they are accompanied by high leakage of the encapsulated drugs [22], leading to restricted applications. In order to better apply liposomes to drug delivery, scientists have developed a series of methods to overcome these shortcomings such as tuning the compositions of phospholipids or adding new components to improve stability and reducing the permeability of liposomes, e.g., for example, increasing the membrane stiffness of the liposomes by incorporating cholesterol [50]. Besides, cationic lipids have been used to further balance the negative charge on the surface of nucleic acid drugs such as siRNA [51], thereby cationic liposomes (CLs) emerged at the required time and were widely studied as a hotspot for gene delivery [52,53]. Generally, CLs are composed of cationic lipids, including 1,2-dioleoyl-3-trimethylammonium-propane (DOTAP), 1,2-dimyristoyl-3-trimethylammonium-propane (DMTAP), N-[1-(2,3 dioleyloxy)propyl]-N,N,N-trimethylammonium chloride (DOTMA), 1,2-dioleyloxy-N,N-dimethyl-3-aminopropane (DODMA), and neutral auxiliary lipids including 1,2- dioleoyl-sn-3-phosphoethanolamine (DOPE) and dioleoyl phosphatidylcholine (DOPC) [54]. Research by Hattori et al. [55] confirmed that the type of cationic lipids has a huge impact on the biological distribution and knockdown efficiency of siRNA in vivo. In other words, the therapeutic effect of CLs is determined by the rational design of lipid composition [56].

**Figure 3:**
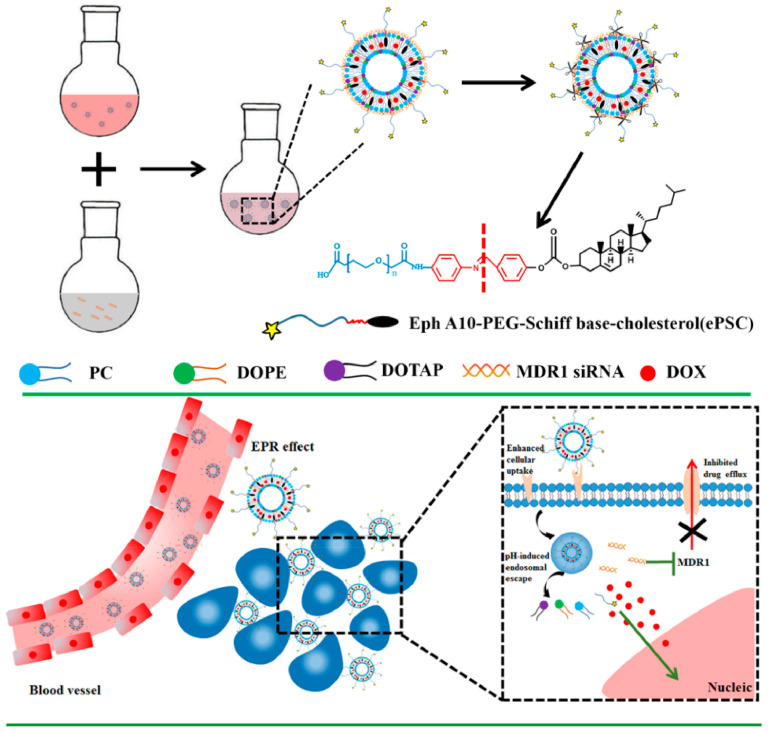
Schematic Illustration of the approach to overcome MDR by multifunctional DOX + siRNA/ePL lipoplexes. Reprinted with permission from Zhang et al. [58]. Copyright (2018) American Chemical Society.

At the same time, with the emergence of multidrug resistance (MDR), it is often difficult for a single chemotherapy drug to fulfil role and their anti-tumor effect. Thus, co-delivery of nucleic acid drugs (e.g., siRNA, miRNA) and chemotherapy drugs (e.g., DOX, PTX, CDDP) through cationic liposomes to reverse drug resistance is increasingly sought after [57]. Zhang et al. [58] have developed liposomal complexes (shortened as DOX +siRNA/ePL) with both pH sensitivity and antibody-mediated targeting, carrying MDR1–siRNA and the antitumor drug DOX (Figure 3). The DOX +siRNA/ePL had high serum stability and showed an incremental uptake by MCF-7/ADR cells and enhanced P-gp downregulation efficacy, demonstrating the excellent potential to overcome the MDR effect. In another report, the use of paclitaxel (PTX) in combination with anti-survivin siRNA in redox-sensitive oligopeptide liposomes was shown to be an effective strategy in treating breast cancer and metastasis. In order to improve the antitumor effect of PTX, Chen et al. [59] designed this formulation to specifically downregulate the overexpression of survivin in 4T1 breast cancer cells to overcome PTX resistance. Mice bearing 4T1 tumors treated with liposomes showed the slowest tumor growth speed to controls, demonstrating the high anti-tumor efficacy of the combination of anti-survivin siRNA and PTX. However, an excessive positive charge was able to interact with a negative charge on the cell surface and destroy the cell membrane, leading to high cytotoxicity in the body [60]. For this, these strategies may face some inevitable problems such as safety and stability [61]. Consequently, developing a safe and efficient CL gene vector is urgent due to the huge demand for biomedicine.

Proper packaging of nucleic acids in liposomes through condensation using polymer materials, such as polyethylene glycol (PEG), can improve the efficiency of drug delivery. Liposomes modified by polyethylene glycol (PEG) are called invisible liposomes; not only does it reduce the rapid clearance of the drug from the reticuloendothelial system and extend the circulation time of the drug in the body, but it can also deliver various drugs to the tumor area effectively and safely [62]. For example, the formulation of patisiran, the first RNAi drug approved by FDA, contained four types of lipids: PEG_2000_-C-DMG, DLin-MC3-DMA, 1,2-distearoyl-sn-glycero-3-phosphocho-line (DSPC), and cholesterol. It delivers the anti-TTR siRNA into the liver, the main TTR producer, via systemic administration. Patisiran enters the liver by binding to ApoE receptors on the surface of hepatic cells. When patisiran navigates in the circulatory system, it is first opsonized by apolipoprotein E (ApoE). The PEGylation provides the optimum circulation time for patisiran and paves the way for the further uptake of it by hepatic cells contained apolipoprotein E receptors [6]. Seraj et al. [63] chose the Eg5 gene as an effective target, constructed and expressed a Eg5 shrna plasmid (pAAV-shEg5), and formed p_shEg5 plasmid/liposome complexes (p_shEg5@LS) using PEGylated DC-Chol/DOPE cationic liposomes. The tumor growth suppression experiment in vivo demonstrated that the p_shEg5@LS lipid complexes showed a longer-lasting anti-cancer effect than the siEg5@LS lipid complexes without PEG modification. Moreover, the results of their research suggested that this lipid system can avoid off-target effects by reducing the dosage of RNAi therapeutic drugs in patients. Lee and Ahn developed a PEGylated liposomal system to deliver siRNA against kinesin spindle protein (KSP) for gene silencing via systemic injection. In this report, PEGylated DC–Chol/DOPE–siRNA lipoplexes exhibited enhanced tumor accumulation compared to non-PEGylated DC–Chol/DOPE–siRNA lipoplexes via less renal excretion and liver clearance and longer circulation time in vivo [64].

#### 4.1.2. Solid Lipid Nanoparticles

As another widely used lipid-based nanoparticle, solid lipid nanoparticles (SLNs) also play an important role in drug delivery (Figure 2b). Solid lipid nanoparticles are sub-particulate drug delivery carriers composed of natural or synthetic high-melting-point lipids that are solid at room temperature and composed of stable and biodegradable lipids or spherical particles with a size of 50–1000 nm [65,66]. Solid lipid nanoparticles have many advantages, such as high bioavailability, the feasibility of large-scale preparation, increasing drug accumulation in cancer cells, and overcoming tumor resistance [67]. Controlling the release of drugs in specific tissues is available via SLNs, since it has significant superiority in targeting specific tissue [66,68,69]. Hence, SLNs show great application potential in drug delivery, and SLNs were created as an alternative to traditional carrier systems like liposomal nanoparticles [70].

**Figure 4:**
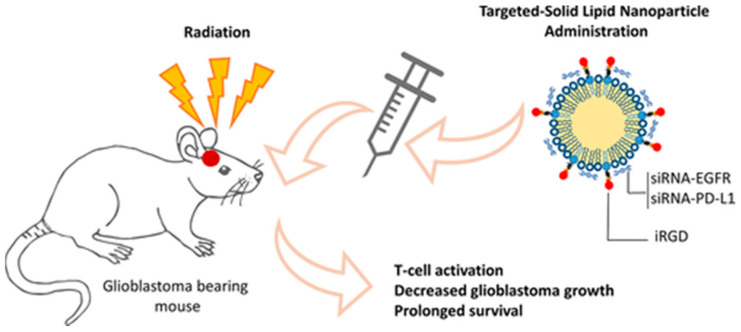
Schematic illustration of the brain tumor therapy. Reprinted with permission from Erel Akbaba et al. [72]. Copyright (2019) American Chemical Society.

From the literature, the fatty acids, monoacylglycerols, diacylglycerols, etc., are usually used as the main lipid components of SLNs. Especially, palmitic acid and stearic acid are compatible with the lipid composition of animal tissues, so they are generally used as the first-choice lipid material for preparing lipid nanoparticles [71]. In a recent study, Erel-Akbaba et al. [72] developed a new type of SLNs using the microemulsion dilution technique (Figure 4). The siRNAs against both epidermal growth factor receptor (EGFR) and PD-L1 were jointly delivered to glioblastoma cells. When systemic administration of targeted SLN after radiation therapy, the drug delivery system can significantly inhibit the expression of EGFR in tumor cells; moreover, it prolonged mouse survival. Solid lipid nanoparticles have excellent delivery effects for low-soluble drugs; therefore, they have been selected as delivery systems for lipophilic anticancer drugs (such as PTX, SN38) [73]. From another recent study, Büyükköroğlu et al. [74] used a solvent emulsification/evaporation method to prepare three kinds of SLNs drug delivery systems: encapsulating Bcl-2 siRNA, paclitaxel, and Bcl-2 siRNA/paclitaxel for the treatment of cervical cancer. The results showed that the therapeutic effect of the combined drug was improved obviously. Bae et al. [75] designed dot-incorporating SLNs for the co-delivery of paclitaxel and Bcl-2-targeted siRNA. The reconstituted low-density lipoprotein (LDL) with a stable core/shell nanostructure was used as a carrier of paclitaxel, and quantum dots were introduced to visualize the intracellular translocation of SLNs into cancer cells. The Bcl-2-targeted siRNA was stably bonded to the outer surface of SLNs by electrostatic interaction. The experiment result showed that both paclitaxel and Bcl-2 siRNA can be delivered into human lung carcinoma cells via the developed solid lipid nanoparticles. However, SLNs also have some inevitable shortcomings, for example, the loading efficiency is very low due to the fact of its own defect in its crystalline structure. In addition, the drug may be discharged at any time under storage conditions [76]. The clinical application of SLN preparation detected is still limited because of the unpredictable security issues; overall, this is a great challenge on how to further understand the formation process, the particle structure, and pharmacokinetic properties of SLNs at the molecular level.

### 4.2. Micellar Nanoparticles

A micellar nanoparticle (Figure 2c) is a self-assembled aggregate particle formed by a surfactant or amphiphilic block copolymers when their concentration exceeds a certain critical value in aqueous solution, and the formed particle is no more than 200 nm in size, generally [77]. Micellar nanoparticles are the most basic colloidal drug carrier. The hydrophilic block extends to the water and forms a hydrophilic shell that can be linked with polyethylene glycol (PEG) to prevent the micelles from non-specific uptake by reticuloendothelial systems (RES), thereby realizing long circulation of drugs in vivo. The hydrophobic block can become a hydrophobic core through intermolecular forces such as hydrogen bonds and van der Waals forces [78,79]. Thus, this unique core–shell structure of micelles presents a potential delivery system for hydrophobic and poor bioavailable compounds and enhances the drug internalization and tissue-specific targeting [80].

Wen et al. [81] developed a new delivery system of micellar nanoparticles modified with angiopep-2 (Ap) to co-deliver VEGF siRNA and paclitaxel (PTX), named the Ap-CSssSA/P/R complex. In vitro and in vivo Ap-CSssSA/P/R complexes showed an excellent silencing effect of VEGF gene, and complexes via LRP1-mediated targeting delivery exerted a higher neovascularization inhibition, compared to naked PTX-loaded nanoparticles. Joshi et al. [82] prepared hypoxia-sensitive micellar nanoparticles based on azobenzene groups for the co-delivery of doxorubicin (DOX) and anti-P-gp siRNA, termed PAPD. Under hypoxic conditions, anti-P-gp siRNA delivered by PAPD showed up to a 60% P-gp downregulation. Recently, a dual pH-sensitive micellar nanodrug that can achieve the codelivery of IKKβ siRNA and STAT6 inhibitor AS1517499 via the M2-targeting peptide was reported by Xiao et al. [83]. The M2-targeting peptides were hidden by the pH-sheddable PEG corona so that the micellar nanodrug could efficiently reduce the immune side effects because of the acidic tumor microenvironment. Besides, the IKKβ siRNA and AS1517499 could synergistically promote the M1 polarization of tumor-associated macrophages (TAMs) with different mechanisms to suppresses tumor growth and metastasis. This year, Norouzi et al. [84] researched another dual-functional polymeric micelle (PM) to solve the limited therapeutic efficiency of anticancer drugs. The PMs with multifunctional tri-layer containing 4-(N)-stearoyl gemcitabine (GemC18), NF-κB siRNA, and tri-block copolymers (PCL–PEI–PEG) were designed to target AsPC-1 (human pancreatic cancer cell line) and MCF-7 (human breast cancer cell line). From this literature, the tri-block copolymers (PCL–PEI–PEG) were beneficial for PMs to electrostatically bind with siRNA and extended blood circulation half-life. As opposed to conventional GemC18 administration, the PMs/GemC18/anti-NF-κB siRNA PMs significantly reduced the value of IC50 after 48 and 72 h of incubation. From these results, there are reasons to believe that co-delivery of siRNA and chemical drugs via micelle nanocarrier would be a promising platform to tumor therapy.

### 4.3. Polymer-Based Nanoparticles

Polymer-based nanoparticles have a wide range of applications in the field of biological preparations, which may be attributed to their versatility in synthesis [85,86]. Especially, these nanomaterials have a responsive ability to stimulation such as enzymes and pH in the body [87,88]. In addition, Li et al. [89] considered that some polymers could even be adjuvants in the carrier structure. There are many types of polymer-based nanoparticles: natural polymers include chitosan, cyclodextrin (CD), and cyclodextrin and synthetic polymers include polyethyleneimine (PEI), polylactic acid (PLA), and dendrimer [90]. Among the materials mentioned above, synthetic cationic materials are preferred in nano-drug delivery. One advantage is that they can be made into controllable sizes and shapes and another point is the nature of their cations; these materials could condense and load anionic siRNA molecules through electrostatic interactions, forming complexes and targeting siRNA to specific disease areas smartly [91].

#### 4.3.1. PEI-Based Nanoparticles

**Figure 5:**
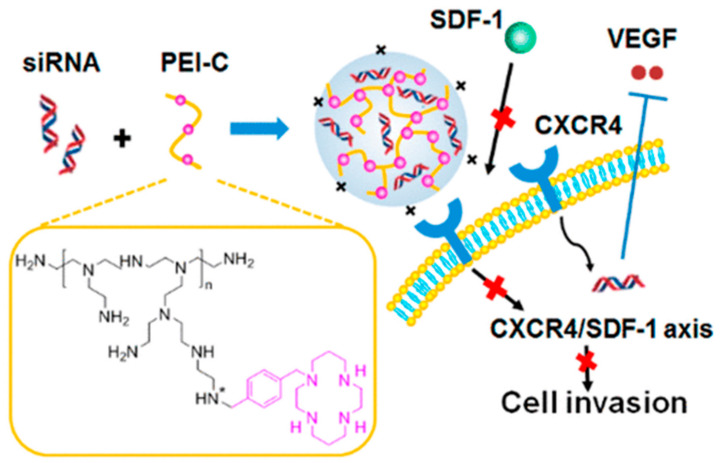
The mechanism of action of PEI-C/siVEGF polyplexes. Reprinted with permission from Zhou et al. [100]. Copyright (2018) American Chemical Society.

Polyethyleneimine (PEI) is one of the most extensively developed cationic polymers and a typical polymer carrier for the delivery of siRNA (Figure 2d). Polyethyleneimine does not only has a good affinity with siRNA [92], more importantly, PEI has the unique proton sponge effect [93], so that PEI is protonated in the body easily and can destroy lysosomes to release siRNA in cells [94]. Many studies have reported that exosomes or ECVs (ECVs can be divided into exosomes) can deliver siRNA [95,96]. Zhupanyn et al. [97] firstly transferred small RNAs by combining PEI-based nanoparticles with ECVs produced by different cell lines. In this experiment, Western blot results showed that the expression of survivin protein decreased by 50% in the group of ECV-modified PEI/siRNA complexes. However, Saw et al. [98] stressed that the abundant charge interactions of PEI also hindered the release of intracellular siRNA and induced cell toxicity. Thus, Yu et al. [99] investigated a system that utilizes the DNA product of rolling circle amplification (RCA) and PEI to co-deliver siRNA. The RCA product could neutralize the strong positive charge of PEI and formed a stable polyplex (PEI/RCA siRNA). Furthermore, Zhou et al. [100] also developed a new PEI derivative to overcome the side effect of the positive charge, which are presented in Figure 5. In this study, the PEI was modified with cycloam, and this system could transfer anti-VEGF siRNA and inhibit CXCR4 at the same time.

**Figure 6:**
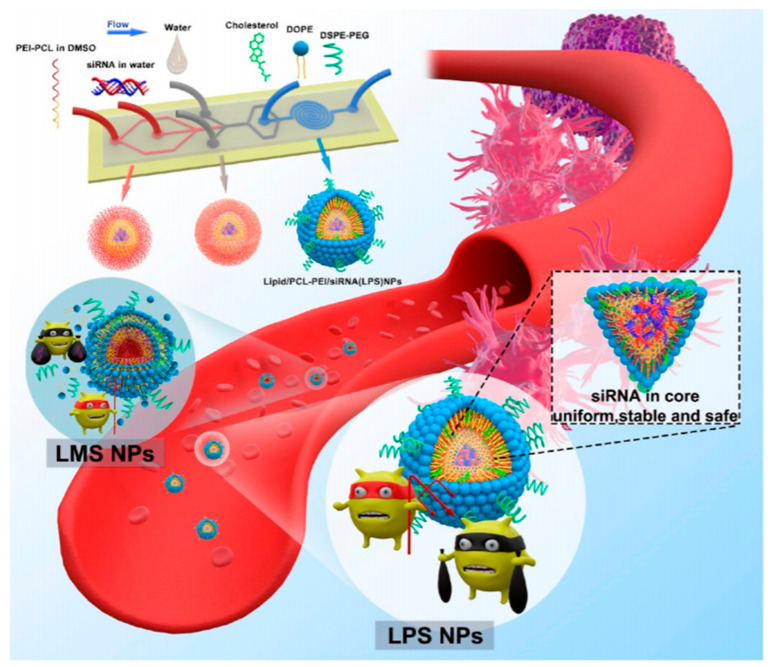
Schematic illustration of the preparation of LPS NPs with the aid of three-stage microfluidic technology. Reprinted with permission from Wei et al. [109]. Copyright (2020) American Chemical Society.

While PEI with a molecular weight of 25 kDa was regarded as the “gold standard” for transfection [92], some studies found that a higher molecular weight (HMW-PEI) means higher transfection efficiency, meanwhile, it induces more serious cytotoxicity in the biological process. On the contrary, low molecular weight (LMW-PEI) has low cytotoxicity but poor transfection activity [101]. Meneksedag-Erol et al. [102] modified LMW-PEI (1.2KDa) with different molecular PrA (a short propionic acid), and it was found that an optimal hydrophobicity/hydrophilicity balance was crucial for siRNA transmission effectively. Therefore, many researchers are committed to modifying the structure of low molecular weight PEI (LMW-PEI) to improve its safety and transfection efficiency and decrease the unnecessary cytotoxicity in vivo. For instance, the surface of PEI can be modified by covalent bonds with PEG [103], polysaccharides [104], and hydrophobic groups [105]. The PEI-polymers based on polysaccharides can improve the half-life of blood circulation and avoid the clearance of reticuloendothelial cells [106]. Park et al. [104] studied low molecular weight PEI grafted hyaluronic acid (HA) to deliver TGF-β siRNA (HA is a glycosaminoglycan abundant in the body [106]). The complex of siRNA/(PEI-SS)-g-HA showed excellent gene silencing efficiency in vitro. In another design, Fan et al. [107] fabricated an intelligent delivery system that consisted of low molecular weight PEI (1.8 kDa) modified by Triton X-100 and 4-carboxyphenylboronic acid (PBA) coupled with dopamine-grafted vitamin E (VEDA). The system could deliver two therapeutic siRNA (siEg5 and siEGFR) to induce RNAi in nude mice. In this thesis, compared with the negative control groups, the gene silencing efficiency of TXPPBA–PEI/VEDA/siRNA complex was notably increased. Ewe et al. [108] and Wei et al. [109] creatively established a new type of lipid−polymer nanoparticle, respectively. In research by Wei et al. [109] (Figure 6), they synthesized LMW–PEI nanoparticles with the aid of microfluidic technology (lipid/PCL–PEI/siRNA) to protect the siRNAs completely by cationic materials. As might be expected, the hybrid nano-assemblies successfully offered a way for siRNA delivery and had an obvious inhibitory effect on tumor growth and no obvious systemic toxicity.

#### 4.3.2. PAMAM-Based Nanoparticles

**Figure 7:**
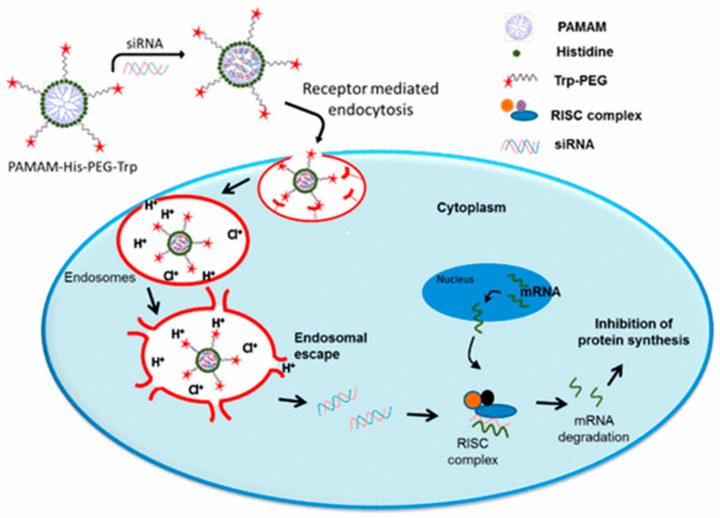
The schematic of cellular uptake mechanisms of PAMAM–His–PEG/siRNA. Reprinted with permission from Tambe et al. [119]. Copyright (2017) American Chemical Society.

At present, polyamide-amine (PAMAM) is one of the most frequently studied dendrimers [110]. Polyamide-amine has the basic characteristics of a dendrimer such as precise molecular structure, hydrophobic cavities in the molecules [111], and relative molecular controllability. With these characteristics, PAMAM can effectively encapsulate nucleic acids and other therapeutic drugs [112] and is an ideal carrier for targeted therapy and diagnostic drugs [113]. Apart from that, PAMAM is generally covered with a large number of cationic primary amine groups; this feature can convert a complex as a whole into nano-scale polymers to increase the absorption of nucleic acid drugs into cells at a physiological pH [114,115]. However, there also exposes a serious problem, the PAMAM-mediated polymer delivery systems are very sensitive to serum in the internal environment, leading to low transfection efficiency and rapid blood clearance. Of all these, it may be ascribed to the strong positive charge from the primary amine group of PAMAM [116]. To mitigate the toxicity of the positive charge, Zhang et al. [117] innovatively prepared mixed dendrimer micelles (MDMs). They synthesized generation 4 polyamidoamine (G4 PAMAM) with PEG2k-DOPE first, then connected it with mPEG2k–DOPE and coated it with hyaluronic acid (HA) to co-deliver MDR-1 siRNA and DOX. Hyaluronic acid can help the micelle shielding the excess positive charge, protect siRNA against enzymolysis from RNase-mediation, and generate stable complexes to encapsulate siRNA. In 2017, Liu et al. [118] prepared a nano-complex with PAMAM dendrimer generation 5.0 (G5) for targeting the MDR-1 gene. The dendriplexes (PAMAM–siRNA) modified by phospholipid (PL) successfully reversed multi-drug resistance (MDR) and impaired over-expression of the p-gp protein. In the another report, Tambe et al. [119] developed a triblock compound (PAMAM–histidine–PEG) to package siRNA. The histidine was considered a proton sponge to increase the transfection efficiency of PAMAM according to Chen et al. [120]. The experimental results suggest that the triblock complex will be a promising treatment for all cancer cells overexpressing LHRH. The process is illustrated in Figure 7. Additionally, PAMAM was also used to modify other inorganic nanoparticles [121,122]. Long et al. [123] synthesized halloysite nanotubes (HNTs) and grafted PAMAM in order to deliver siVEGF for the breast cancer model. The complex could effectively inhibit the growth of tumor cells and reduce the expression level of the VEGF gene in tumor cells.

#### 4.3.3. Noncationic Polymer Nanoparticles

Generally, siRNA is negatively charged, so cationic nanoparticles are usually selected to compress it with to form the delivery systems that can contribute to the uptake of nanocomposites by cells [124]. Nevertheless, the high charge of cationic nanoparticles may result in more toxicity to normal cells, although they have the high siRNA loading efficiency [125]. In this case, noncationic materials (Figure 2f) may be a better choice. A new type of spherical nucleic acid (SNA) nanocarrier that can be taken up by cells efficiently without significant cytotoxicity and immunogenicity has been reported in spite of its negative charge [126]. Inspired by this, Ding et al. [127] employed cross-linked nanogels with a negative charge as the carrier of siRNA. In detail, a DNA-grafted polycaprolactone (DNA-gPCL) was designed to form spherical and nanosized hydrogels with SNA architecture using siRNAs as the cross-linkers. The siRNA was completely embedded and protected. Besides, the crosslinked nanogels exhibited remarkable physiological stability and thermostability and exhibited significant gene silencing efficiency as well as excellent inhibition of tumor growth in vivo and in vitro. In 2018, Jiang et al. [124] provided a supramolecular strategy for RNA delivery with low cytotoxicity. In this research, a methacrylate random copolymer P1 was used to bind RNA via electrostatic interaction. Then, the complex self-crosslinked subsequently to trap dsRNA inside the nano-assembly by adding dithiothreitol (DTT), and most of the cationic moieties were eliminated at the same time. Compared to classical cationic delivery vehicles, this non-cationic RNA deliver strategy can reduce cytotoxicity substantially.

### 4.4. Gold Nanoparticles

**Figure 8:**
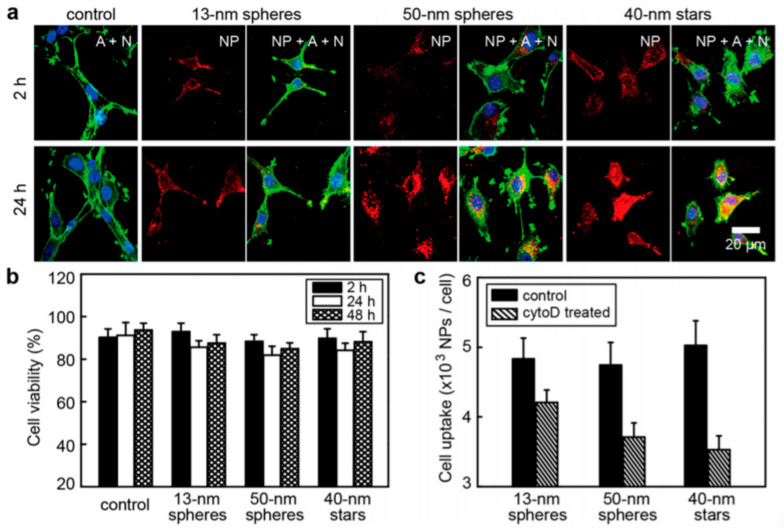
The anti-tumor activity of three NPs in vitro and cell morphology changes induced by larger constructs. (**a**) Confocal microscopic images of U87 cells treated with PBS (control) or three NPs (0.2 nM). (**b**) The inhibitory effect of three NPs (0.2 nM) on U87 cells. (**c**) Effect of CytoD on the uptake of three NPs by U87 cells. Reprinted with permission from Yue et al. [132]. Copyright (2017) American Chemical Society.

Gold nanoparticles (AuNP) (Figure 2g) are a material with a size of less than 100 nm at least one dimension [128]. It can be made into different shapes and sizes, for instance, nanospheres, nanowires, nanorods, nanoshells, and nanocages [129]. Almost all types of gold nanoparticles have low cytotoxicity and a preeminent ability to resist the degradation of enzymes in vivo [130,131]. Even though a recent paper found that the different shapes and sizes represented the different distribution of AuNPs in the body [132]. Yue designed different formulations of siRNA–gold nanoparticles including 13 nm spheres, 50 nm spheres, and 40 nm stars. The experimental results of confocal fluorescence images (Figure 8a), cell viability (Figure 8b), and cell uptake (Figure 8c) are represented in Figure 8. It can be seen from the figures that the larger particles (50 nm spheres and 40 nm stars) revealed higher transfer efficiency for siRNA. Moreover, in the study from Morgan et al. [133] there were three different shapes, yet they were the same three ~45 nm diameter gold nanoparticles. By side-by-side comparison of siRNA loading and gene knockout, the nanoshells and nanocases displayed the higher downregulation of GFP expression than nanorods. It confirmed that different shapes of nanoparticles have a significant impact on the delivery of gene-related drugs even if they are of the same size.

Furthermore, AuNPs exhibit prominent optical properties, which are derived from surface plasmon resonance (SPR). At present, the characterization of medical applications and biological activity for AuNPs are mostly relying on the SPR [134]. For example, Liu et al. [135] constructed a nanoplatform for lung cancer model based on SPR characteristics, and they adopted gold-based nanoprisms (GNPs) loaded with human hPD-L1 siRNA, coated by negatively-charged PSS, and positively-charged PDADMAC to improve biocompatibility and stability (formed GNPs–hPD-L1 siRNA). Hitherto, PD-1 (programmed cell death protein 1) has been found as a receptor protein on the surface of T cells, able to interact with PD-L1 (PD-1 ligand) expressed on the surface of tumor cells and causes the immune escape of cancer cells [136,137]. In this anti-tumor study, the viability of HCC827 cells treated by GNPs–hPD-L1 siRNA nanoprisms with laser irradiation was significantly less than the group of GNPs–hPD-L1 siRNA without laser irradiation. Of course, in addition to gold, metals, such as silver, platinum, and copper, are also used to develop metallic nanocarriers [138].

### 4.5. Mesoporous Silica Nanoparticles

**Figure 9:**
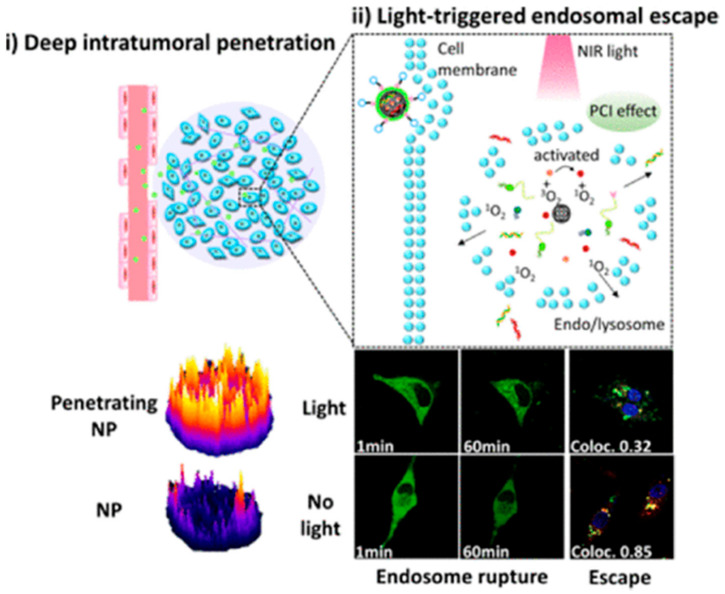
Schematic Illustration of light-triggered RNA delivery by tumor-penetrating iMSNs for siPlk1/miR-200c combination therapy. (**i**) Deep intratumoral penetration. (**ii**) Light-triggered en-dosomal escape. Reprinted with permission from Wang et al. [152]. Copyright (2019) American Chemical Society.

Currently, researchers see silicon as more promising than other nanomaterials in biomedical applications including bioimaging and disease treatment [139]. Compared with “soft” materials, such as liposomes or polymers, it has been demonstrated that silicon-based materials have higher loading capacity [140]. Mesoporous silica nanoparticles (MSNPs) (Figure 2h) stand out among all-silicon materials with excellent physical and chemical properties, which reveal the formidable advantages as new inorganic materials for biomedical applications [141]. For instance, Cao et al. [142] proved that MSN is a promising photothermotherapy carrier for inhibiting the proliferation of tumors. On one side, the adjustable pore size is beneficial for increasing drug loading and controlling the rate of drug release [143]. Similarly, the particle size of MSNs affects drug release. Bouchoucha et al. [144] synthesized MSNs with different particle sizes from 45 nm to 500 nm. They found that the smallest nanoparticles (45 nm) had a much higher cellular uptake efficiency and enhanced the release of DOX in the tumor. Drugs, on the other side, can be integrated into both channels and surfaces of MSNs by electrostatic adsorption or covalent bonding [145,146]. In addition, MSNs have been proved to be safe and biodegradable in vivo in a large number of animal experiments [147]. The MSNs, a versatile and ideal nanocarrier, can load small molecule chemotherapy and gene drugs for cancer therapy including DOX hydrochloride [148], cisplatin [149], DNA [150], and siRNA [151]. Wang et al. [152], reported a mesoporous silica nanoparticle (iMSNP) co-delivery dual-type therapeutic RNAs (siPlk1 and miR-200c). Moreover, they utilized both photosensitizer indocyanine green (ICG) and penetrating peptide iRGD to modify and encapsulate the MSNPs (Figure 9). The result of the experiment showed that the iMSN/Plk1 + 200c + ICG (+light) group revealed higher inhibition ratio than other groups (iMSN/Plk1 + 200c + ICG (−light), MSN/Plk1 + 200c + ICG, iMSN/NC + ICG, iMSN/Plk1 + NC + ICG, iMSN/200c + NC + ICG) in tumor cell proliferation of orthotopic breast cancer model. The MSNPs, with photodynamic therapy developed above, have substantial achievements for siRNA–miRNA combination to cure cancer and provide a novel idea to deliver gene drugs.

In general, the versatility of MSNs can be improved by surface functionalization via various types of polymeric materials such as PEG, PEI, and PAMAM [153]. The silica polymer core/shell nanohybrids will enhance transfer efficiency and have a huge improvement particularly in controlled drug delivery [154]. Li et al. [155] described a siRNA delivery system (M-MSN_VEGF siRNA@PEI-KALA), the magnetic MSNs (M-MSNs) were functionalized by PEI and peptide (KALA). With fusogenic peptide Kala, nanoparticles can pass through the cell membrane into the tumor cells, then efficiently escape from the endolysosomes to release the loaded siRNA molecules through the proton sponge effect of PEI. The next year, their group created another M-MSNs [156], the new nanoparticles (M-MSN_VEGF siRNA@PEI-PEG-KALA) modified with PEI, PEG, and peptide (KALA) simultaneously allowed siRNA to enter the mesopore of M-MSNs. The platform further prolonged the half-life of drugs in the blood stream and improved survival. Thus, this nanosystem is considered a promising platform for gene delivery. In 2019, Xie et al. [157] first reported a hybrid nano-complex with N9 peptide and DOX against Bcl-2-positive cancer cells in vitro and in animals; the MSNs connected highly branched polyamidoamine (PAMAM) showed a strong synergistic anticancer effect. These organic–inorganic hybrid MSNs have a significant impact on the development of cancer therapy.

Some researchers have also coupled some aptamers to MSNs for improving drug targeting. Yang et al. [158], designed MSNs with targeting molecules (Sgc8), this nanoparticle-loaded DOX can selectively enter the desired tissue and kill the tumor cells continuously. In another recent study, Han et al. [159] prepared MSNs modified by TAT peptide and can layer-by-layer self-assembly to co-deliver DOX and siVEGF. The multi-layered nanocomplexes successfully delivered siRNA and DOX to the cytosol and nucleus, respectively.

### 4.6. Iron Oxide Nanoparticles

Iron oxide nanoparticles (IONPs) (Figure 2i) are inorganic nanomaterials with great targeting ability, superparamagnetism, and suitable size [160]. The IONPs have been approved by the FDA as use as a contrast agent and then spawned a new research field called magnetic resonance imaging (MRI) [161,162]. Fluorescent dyes, tumor targeting molecules and chemotherapeutic drugs can bind to IOPNs to achieve the integration of tumor targeting diagnosis and treatment [163]. Recently, Zhang et al. [164] constructed a gene therapy approach by using IONPs for the postoperative treatment of glioblastoma patients. The results showed that the IONPs as the efficient ferroptosis and apoptosis inducers are safe for the treatment of glioblastoma. In another study, the tumor therapy ability of superparamagnetic iron oxide (SPIO) nanoparticles and poly (propyleneimine) generation five dendrimers (PPI G5) siRNA co-delivery system was evaluated by Taratula et al. [165]. The great targeting capability and anti-tumor activity of the co-delivery system were showed in vitro experiment. In this research, SPIO as the contrast agent and delivery vector provided a new paradigm for the development of targeted multifunctional siRNA vector and real-time monitoring of RNA interference therapeutic responses.

### 4.7. Upconversion Nanoparticles

**Figure 10:**
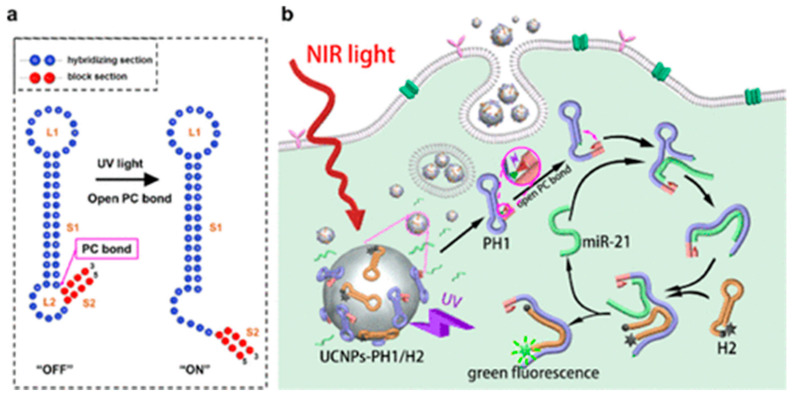
The principle of controllable miRNA imaging nanoprobe. (**a**) The structure of the PH1 probe in this experiment. (**b**) The principle of nanoprobes working in cells [170]. Copyright (2020) American Chemical Society.

Upconversion nanoparticles (UCNPs) (Figure 2j) are known for their excellent optical properties and have developed the field of biophotonics in combination with optical bioimaging technology [166]. The UCNPs doped with rare earth elements can radiate the high-frequency photon nanometer particles when excited by two or more low-frequency photons. This process violates Stokes law, so it is also called “anti-Stokes luminescence” [167]. The luminescence mechanism of UCNPS is mainly divided into three categories: excited state absorption (ESA), energy transfer upconversion (ETU), and cooperative sensitization upconversion (CSU) [168]. Unfortunately, UCNPS-based sensors have been found to have poor sensitivity after signal amplification and low quenching efficiency in many studies [169]. These issues may be addressed by a novel NIR-activated nanoprobe (Figure 10). Zhao et al. [170] proposed a nanoprobe as NIR-to-UV converter that can be used for controllable imaging of miRNA in vivo. It integrated the advantages of UCNPs and UV-responsive beacon probes to improve the efficiency of optical imaging remarkably. We are convinced that this NIR-activated UCNP strategy will be effectively used in biophotonics, especially in cancer treatment.

Great progress has been made in the research of nanoparticles for the delivery of RNAi molecules showing the promising future of this field. The advantages and disadvantages of these nanomaterials are briefly described in Table 3 below. For the delivery of siRNAs, we found fewer inorganic nanoparticles in clinical studies compared to organic nanoparticles. Actually, there is no unified conclusion on the biodegradability and biocompatibility of inorganic nanoparticles and the results of different studies are always contradictory. Recently, a gold nanoparticle-based RNAi drug (NU-0129) for the treatment of glioblastoma (GBM) is in early phase 1 experiment. The results of the study in non-human primates and human phase 0 clinical trials showed the safety of NU-0129 with systemic administration [171,172]. We expect that this delivery system can break the inherent shortcomings of inorganic nanoparticles and send the drug from bench to bedside. Human biological system is extremely complex so the interaction between nanoparticles and proteins or other biological components may lead to unique biological distribution, immune response and metabolism. Therefore, it may be necessary to carefully evaluate the long-term toxicity and biodegradability of nanoparticles-based RNAi therapeutic before clinical trials.

## 5. Conclusions

Present treatment strategies for cancer, malignant diseases that plague the world, still have many limitations. There have been a large number of reports demonstrating that RNAi-mediated gene silencing has a significant inhibitory effect on tumor cells. As a consequence of targeting specific genes and having extremely high silencing efficiency, siRNA has few side effects. Nanoparticles have many distinct features, such as suitable size, and can be modified by active targeting molecules. Over the last decades, the research on nanoparticles has been revolutionized. Thus, nanoparticles as the protective carrier for the systemic delivery of siRNA can contribute to the development of RNAi therapy, due to the instability and low targeting of naked siRNA in blood circulation. Despite current successes, there are still challenges that hinder the clinical application of RNAi-based cancer treatment so that the development of it is still in the preclinical trial. Degrading in circulation, the low uptake efficiency of siRNA by tumor cells, and the off-target effects are the obstacles that scientists are trying to eliminate in the systemic administration of RNAi-based cancer therapeutics. In the near future, RNAi-based therapy will become an important means in the clinical practice of cancer treatment.

## Figures and Tables

**Table 1:** Clinical trials with RNAi-based therapies (Available online: https://clinicaltrials.gov, accessed on 20 November 2020).

Name	Target	Disease	Phase	Carrier	Status	Ref.
Mesenchymal stromal cell-derived exosomes with KRAS G12D siRNA	KrasG12D	Metastatic pancreatic adenocarcinoma, Pancreatic ductal adenocarcinoma, Stage IV Pancreatic Cancer AJCCv8	Ⅰ	Exosome	Not yet recruiting	[10]
EphA2-siRNA	EphA2	Advanced malignant solid neoplasm	Ⅰ	Liposomes	Recruiting	[11]
Atu027	PKN3	Advanced solid tumors, Carcinoma, Pancreatic Ductal	Ⅰ Ⅱ	Liposomes	Completed Completed Completed Completed	[8] [12] [13] [14]
CALAA-01	RRM2	Solid tumors	Ⅰ	Cyclodextrin polymer-based NPs	Terminated	[15]
DCR-MYC	MYC	Hepatocellular Carcinoma, Solid tumors, Multiple myeloma, non-Hodgkin’s lymphoma, pancreatic neuroendocrine tumors, PNET, NHL	Ⅰ II	Lipid nanoparticle	Terminated	[16,17]
TKM-080301	PLK1	Hepatocellular Carcinoma Hepatoma Liver cancer, Adult liver cell Carcinoma, Adultneuroendocrine tumors, NET, Adrenocortical carcinoma, ACC, Colorectal cancer with hepatic metastases, Pancreas cancer with hepatic metastases, Gastric cancer with hepatic metastases, Breast cancer with hepatic metastases, Ovarian cancer with hepatic metastases	Ⅰ II	Lipid nanoparticle	Completed	[18] [19] [20]
siG12D LODER	KRAS	Pancreatic ductal adenocarcinoma, Pancreatic cancer	Ⅰ	Miniature biodegradable polymeric matrix	Completed	[21]

**Table 2:** Comparison of general properties among siRNA, miRNA, and shRNA.

Properties	siRNA	miRNA	shRNA
Source	Chemically synthesized; Processed from long dsRNA or Pre-shRNA	Endogenic; chemically synthesized; expressed from miRNA vector	Expressed from shRNA vector
Structure	Double stranded; 21–24 nucleotides	18–25 nucleotides; a characteristic two-nucleotide 3′ overhang	Pre-shRNA cleaved by Dicer to obtain the structure similar to siRNA
mRNA target	Single	Multiple	Single

**Table 3:** A summary of the advantages and disadvantages of different nanoparticles.

Nanoparticles		Advantages	Disadvantages	Reference
Organic	Lipid-Based Nanoparticles	(a) Offer wide options of polymer materials (b) Cationic lipids enhance stabilization by electrostatic interactions (c) Easy scale up and manufacturing	(a) Insufficient drug loading (b) Faster drug release (c) RES clearance (d) Short half-life in serum (e) Dose-dependent toxicity	[90,173,174]
	Micellar Nanoparticles	(a) Less toxicity. (b) Promoteblood circulation (c) Hydrophobic cores are favorable for encapsulation of hydrophobic drugs. (d) Biocompatible and biodegradable materials	(a) Immature drug release (b) Low drug loading capacities, (c) Burst release	[173,175,176]
	Polymer-Based Nanoparticles	(a) Water-solubility (b) Biocompatible and biodegradable (c) No or littleimmunological response (d) Unique proton sponge effect (PEI) (e) Easily fine-tuned for any size	(a) Cationic polymers cause cytotoxicity (b) Poor clinical delivery (c) Low efficacy	[5,92,173,176,177]
Inorganic	Gold Nanoparticles	(a) Photosensitive (b) Customizable size (c) Thermal ablation of cancer cell (d) SPR phenomenon	(a) High cost (b) (RES clearance (c) Certain sizes show lethal toxicity (d) Poor targeting ability	[134,151,178,179,180,181]
	Mesoporous Silica Nanoparticles	(a) Adjustable aperture size (b) Large specific surface area offer high drug loading (c) Excellent mechanical stability (d) Easily functionalized (e) Excellent biocompatibility (f) Photothermotherapy	(a) Drug leakage (b) Toxicity to liver (c) Limited blood circulation half-lives	[173,182,183,184]
	Iron oxide Nanoparticles	(a) Superparamagnetism (b) Magnetic targeting (c) Tunable surface modifications (d) MR imaging (e) Low cost	(a) Poor solubility (b) Lack long-term biosafety	[7,185,186,187,188,189]
	Upconversion Nanoparticles	(a) Excellent optical properties (b) Capability of being luminescent probes (c) Deeper tissue penetration (d) Negligible autofluorescence	(a) Immunological toxicity (b) Fluorescence quenching effect (c) Low reproducibility (d) Potential long-term toxicity and unclear systematic clearance	[13,190,191,192]
